# Retinal channelrhodopsin-2-mediated activity in vivo evaluated with manganese-enhanced magnetic resonance imaging

**Published:** 2010-06-09

**Authors:** Elena Ivanova, Robin Roberts, David Bissig, Zhuo-Hua Pan, Bruce A. Berkowitz

**Affiliations:** 1 Department of Anatomy and Cell Biology, Wayne State University, Detroit, MI; 2 Department Of Ophthalmology, Wayne State University, Detroit, MI

## Abstract

**Purpose:**

Ectopic expression of light-sensitive proteins, such as channelrhodopsin-2, represent a novel approach for restoring light-detection capabilities to degenerated retina. A noninvasive method that can detect light-mediated activities of such light-sensitive proteins in the retina in vivo would be important for correlating expression patterns and retinal function. In this study, we tested the hypothesis that retinal uptake of manganese, measured noninvasively with manganese-enhanced magnetic resonance imaging (MEMRI), is a biomarker of channelrhodopsin-2-mediated activity in vivo.

**Methods:**

The eyes of 3-month-old *rd1/rd1* mice were either untreated (“uninjected,” negative control) or injected intravitreally with either saline (“saline,” negative control) or adeno-associated virus carrying a fusion construct of channelopsin-2 (Chop2) and green fluorescent protein (GFP; “Chop2-GFP”). MEMRI examination was performed 2 months later on either dark or continuous bright blue light-exposed mice to assess the distribution and extent of manganese uptake in the retina and optic nerve. In separate experiments, MEMRI was used to map laminar accumulation of manganese vertically through the retina. For comparison, Chop2-GFP expression was evaluated in whole mounts and vertical sections of virus-infected retinas and optic nerve.

**Results:**

In the two control groups (regardless of lighting exposure) and between the control groups and the dark-exposed virus-treated eyes, retinal and optic nerve uptake of manganese did not differ. In light-exposed virus-treated eyes, manganese uptake in the retina and optic nerve was significantly greater relative to the other groups. In a retinal cross-section, manganese accumulation in light-exposed virus-treated eyes was spatially matched with Chop2-GFP expression in the optic nerve and all remaining retinal layers except the inner nuclear layer.

**Conclusions:**

First-time evidence is presented indicating the usefulness of measuring intraretinal manganese accumulation as a noninvasive biomarker of channelrhodopsin-2-mediated activity in vivo.

## Introduction

The death of photoreceptors in retinal degenerative diseases, such as retinitis pigmentosa, results in vision loss and blindness. A new strategy for treating such retinal degeneration morbidity involves restoring light sensitivity in the retina by expression of microbial rhodopsins, such as channelopsin-2 (Chop2), in surviving inner retinal neurons [1]. Channelrhodopsin-2 (ChR2, Chop2 with attached chromophore) is a directly light-gated nonselective cation channel [2]. Viral-based gene transfer is a promising tool for the delivery of transgenes to nondividing mammalian neurons. Following intravitreal injection of recombinant adeno-associated virus serotype 2, ChR2 expression is predominately observed in the retinal ganglion cell and inner plexiform layers, with additional expression in amacrine and horizontal cells, as well as in the optic nerve [1,3,4]. ChR2-mediated light sensitivity in a homozygous rd1 (*rd1/rd1)* mouse, a mouse model of retinal degeneration, has been shown in vitro through electrophysiological recording and in vivo with visual evoked potential recordings [1,5] and visual behavioral testing [6,7]. For further refining this treatment approach, the development of a noninvasive method to detect the ChR2-mediated activity in the retina in vivo would be desirable for correlation of expression patterns of ChR2 and retinal function.

At present, the only noninvasive method available for measuring regional retinal ion regulation in vivo is manganese-enhanced magnetic resonance imaging (MEMRI) [8–12]. In biologic systems manganese is known to behave like calcium ions, which are allowed to enter cells through calcium pathways, such as voltage-gated calcium channels [13,14] and *N*-methyl-D-aspartate receptor [15]. After systemic injection of a modest and nontoxic amount of MnCl_2_, manganese is a surrogate biomarker of normal and diseased retinal function [8–12]. ChR2 channels have been shown to be permeable for calcium ions [2]. However, the applicability of using MEMRI to investigate ChR2-induced activity has not been examined. In this study we test the hypothesis that in a retinal degeneration model (*rd1/rd1* mouse), inner retinal uptake of manganese, measured noninvasively with MEMRI, is a functional biomarker of ChR2-mediated activity in vivo.

## Methods

### Animals and viral injection

All of the animal experiments and procedures were approved by the Institutional Animal Care and Use Committee at Wayne State University and were performed in accordance with the National Institutes of Health (NIH) Guide for the Care and Use of Laboratory Animals. The MEMRI experiments were initially performed using a 4.7T MRI system (Bruker Avance, Billerica, MA) to measure intraretinal signal intensities. When a new 7T system (Bruker Avance) became available, we also performed additional experiments to take advantage of the relatively improved signal-to-noise ratio to measure the spin-lattice relaxation time (T_1_), a parameter whose inverse is more directly related to retinal manganese level than signal intensity [16].

Three-month-old *rd1/rd1* mice (purchased from the Jackson Laboratory, Bar Harbor, ME) received either no intravitreal injection (“uninjected,” negative control, n=3 [dark], n=3 [bright blue light]), an intravitreal injection of saline vehicle (“saline,” negative control, n=3 [dark], n=3 [bright blue light]), or an intravitreal injection of a recombinant adeno-associated virus serotype 2/2 vector carrying a fusion construct of channelopsin-2 (Chop2) and green fluorescent protein (GFP) under a hybrid cytomegalovirus early enhancer and chicken β-actin (CAG) promoter (n=6 [dark], n=6 [bright blue light]) [1]. These groups were studied on the 4.7T system (see below). A smaller subset of animals (n=4, bright blue light [one eye Chop2-GFP, the other uninjected]) were also investigated on the 7T system (see below).

Viral vectors were packaged and affinity purified by the Gene Therapy Program at the University of Pennsylvania. The intravitreal injection procedure has been previously described [2]. Briefly, a subset of 3-month-old mice (C3H/HeJ or *rd1/rd1*) was anesthetized by intraperitoneal injection of a mixture of ketamine (120 mg/kg) and xylazine (15 mg/kg). Under a dissecting microscope, a small perforation was made in the temporal sclera region with a needle. The solution (saline or viral vector suspension in saline [6×10^12^ genome copies (GC)/ml, 1 μl total volume]) was injected into the intravitreal space through the hole with a Hamilton syringe. In some cases, injections were performed in both eyes. All virus-injected and control (uninjected and saline injected) mice were housed in normal 12-h-cycled laboratory lighting.

### High-resolution magnetic resonance imaging experiments

In all cases MEMRI measurements were performed 2 months after intravitreal injection. Before the MEMRI measurement, MnCl_2_ (Sigma, St. Louis, MO) was injected intraperitoneally (ip; 66 mg/kg) on the right side of each awake mouse [12]; mice were maintained in either dark or continuous bright blue light for 4 h. The bright blue light for the first group was generated by high-power light-emitting diodes (LEDs; LXHL-FR5C; Lumileds Lighting, San Jose, CA) with a peak wavelength of 455 nm and light intensity of 1.2×10^16^ photons cm^−2^ s^−1^; these were mounted at the top the animal cage. Before light exposure, atropine (1%; Bausch and Lomb, Tampa, FL) was applied to the cornea of all animals to dilate the pupils.

MEMRI examination was performed on a 4.7T Bruker Avance system to assess manganese uptake horizontally around the optic nerve head. The 7T Bruker Clinscan was used to evaluate the vertical distribution of manganese through the retina. Immediately before each MRI experiment, mice were anesthetized using urethane (36% solution, ip, 0.083 ml/20 g animal weight, prepared fresh daily; Aldrich, Milwaukee, WI). To maintain each mouse's core temperature, a recirculating warm water blanket was used; rectal temperatures were monitored in the magnet using a fiberoptic thermometer (Luxtron, Santa Clara, CA) [17]. In most mice, left and right eyes were separately examined using a 1.0-cm diameter coil.

On the 4.7T, we used an adiabatic pulse sequence [18] to collect a single transverse slice that was positioned in the middle of the lens and middle of the optic nerve (i.e., through the center of the eye). High-resolution images were acquired using an adiabatic spin-echo imaging sequence (repetition time [TR] 350 s, echo time [TE] 16.7 ms, number of acquisitions [NA] 16, matrix size 256×512, slice thickness 620 μm, field of view 12×12 mm^2^, 27 min/image) [18]. These conditions provided 23.4 μm intraretinal resolution near the optic nerve head. On the 7T, a body coil transmission/surface coil reception arrangement was used. After positioning a medial slice (as above), partial saturation T_1_ data were acquired (TE 13, matrix size 160×320, slice thickness 600 μm, field of view 7×7 mm^2^). At each TR, several single images (in parentheses) were acquired in the following order: TR 0.15 s (6), 3.5 s (1), 1.0 s (2), 1.9 s (1), 0.35 s (4), 2.7 s (1), 0.25 s (5), 0.5 s (3). The virus-injected eye was scanned first in two of the four animals studied on the 7T system.

### Magnetic resonance imaging data analysis

#### 4.7T data

Retinal and optic nerve (in a small region-of-interest immediately posterior to the retina) signal intensities were normalized to the signal intensity of the same extraocular muscle in each animal to minimize differences in systemic manganese handling. Note that the data collection was optimized for retinal acquisition and not optic nerve acquisition. In some cases, an oblique slice prevented observing and thus prevented analyzing the optic nerve so that the number of eyes used in the optic nerve analysis was somewhat smaller than that for the retinal analysis. The muscle intensities were measured from a fixed-size region-of-interest drawn in the anterior-most aspect of the inferior rectus muscle. For visualization and comparison purposes, in-house written software was used to map the in situ image into a linear representation for each retina. First, the vitreoretinal border and optic nerve were manually defined. A high-order polynomial was fit to the vitreoretinal border. Retinal data was oversampled along lines perpendicular to the polynomial, and reconstructed into the linearized image, with the optic nerve head as its origin [19]. Within each group, linearized retinas were averaged into a composite image. For quantitative analysis, signal intensities were analyzed using the program NIH IMAGE and derived macros. Vertically, retinal signal intensity data were extracted at 3 pixels posterior to the clearly defined vitreoretinal division (approximately at the level of the inner nuclear layer [INL]). This location was chosen based on our previous work in nondegenerated retinas, the inner retina of the *rd1/rd1* mice in this study which has normal thickness, and results from 7T vertical mapping. Horizontally, pixels were extracted between 0.4 and 1 mm on either side of the optic nerve (i.e., superior and inferior retina) and were analyzed. Intraretinal signal intensity was analyzed using IMAGE (a public domain image processing and analysis program) and derived macros. We controlled for changes in receiver gain differences between animals by normalizing the signal intensity of a fixed region of noise in each rat to a fixed value. Other tissues within the sensitive volume of the coil demonstrated enhancement after manganese injection and thus were considered inadequate as internal references. Inner and outer signal intensity data were extracted and analyzed [12]. Initial efforts to compare the 4.7T data of vertical manganese distribution to the distribution of Chop2-GFP expression ex vivo was limited by an apparent homogenous signal from the entire retina (data not shown). Since the combination of higher magnetic field and T_1_ mapping can provide spatial clarity over a signal intensity analysis [20], T_1_ studies were performed on the 7T system using a small subset of conditions in a paired-eye design (see above).

#### 7T data

Single images acquired with the same TR were registered (rigid body), then averaged. These averaged images were then registered across TRs, and retinas were linearized as before [19]. Using the linearized data, 1/T_1_ maps were generated by fitting a three-parameter T_1_ equation (y=a + b[exp(-cTR)], where a, b, and c are fitted parameters) on a pixel-by-pixel basis using R (v.2.9.0, R Development Core Team 2009) scripts developed in-house and the minpack.lm package (v.1.1.1, Timur V. Elzhov and Katharine M. Mullen minpack.lm: R interface to the Levenberg-Marquardt nonlinear least-squares algorithm found in MINPACK. R package version 1.1–1.). The reciprocal (1/T_1_) maps directly reflect manganese levels [16]. Between eyes, linearized maps were aligned using an automated x–y registration (align slices in stack plugin of ImageJ), and average virus injected or noninjected parameter maps were derived.

### Analysis of channelopsin-2 expression

The expression of Chop2-GFP in the retina was studied in whole mounts (i.e., a horizontal analysis) and in vertical sections following 4 h of bright blue light exposure. Immediately after MRI examination anesthetized mice were decapitated, superior/ inferior eye orientation externally marked, and the eyes were enucleated. The retinas were fixed in the eyecups with 4% paraformaldehyde in 0.1 M phosphate buffer (PB) for 20 min. For the cryostat sections the retinas were cryoprotected with all three solutions starting with 10%, then 20%, and finally 30% weight/volume in PB, which called sucrose gradient and sections were cut at 20 μm. For the whole mounts, the retina was dissected free in PB solution, flat mounted on slides, and coverslipped.

GFP fluorescence, without antibody enhancement, was sufficient to visualize most of the GFP expressing cells in whole mounts and retinal sections.

For double labeling, retinal sections were blocked for 1 h in a PB solution that contained 5% Chemiblocker (membrane-blocking agent; Chemicon, Temecula, CA), 0.5% Triton X-100, and 0.05% sodium azide. The primary antibodies were diluted in the same solution and applied overnight followed by secondary antibodies for 2 h. All steps were performed at room temperature. The primary antibodies were rabbit anti-GFP antibody conjugated to Alexa488 (1:2,000; Molecular Probes, Eugene, OR) and mouse anti-calbindin antibody (1:5,000; Sigma, Saint Louis, MO). Secondary antibodies for calbindin were donkey antimouse Alexa555 (1:600; Molecular Probes).

All images were acquired using a Zeiss Axioplan 2 (Zeiss, Oberkochen, Germany) microscope with the Apotome oscillating grating to reduce out-of-focus stray light. Z-stack images were captured using a Zeiss Apotome microscope with a fixed exposure time. Image projections were made by averaging individual z-stacks of optical sections into a single image, unless otherwise indicated. The brightness and contrast were maximized for each average image using Adobe Photoshop CS4 (Adobe systems incorporated, New York, NY). For comparison, differential interference contrast images were also collected.

### Statistical analysis

Comparisons of MEMRI retinal signal intensities within and between groups were performed using a generalized estimating equation approach [12,21]. A generalized estimating equation performs a general linear regression analysis using all of the pixels in each subject and accounts for the within-subject correlation between adjacent pixels. In all cases, p<0.05 was considered statistically significant. Data are presented as mean±standard error of the mean (SEM) unless otherwise noted.

## Results

### Horizontal analysis

**Figure 1:**
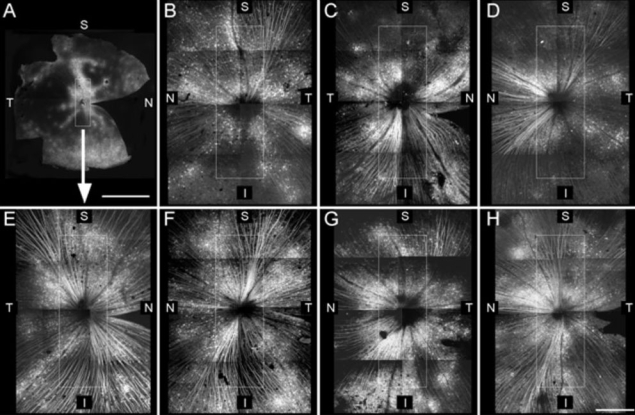
Whole-mount retinas injected with channelopsin-2 viral constructs. **A**: A representative whole-mount retina with channelopsin-2 (Chop2-GFP) expression is shown at low resolution. A white rectangle frames the area around the optic nerve head that was analyzed in the manganese-enhanced magnetic resonance imaging (MEMRI) study; this area is shown magnified in **E**. **B-H**: Expression of Chop2-GFP was studied in montaged and magnified images of the retinas of dark-exposed mice (**B-D**) and light-exposed mice (**E-H**). Scale bars are 2 mm in **A** and 0.5 mm in **B-H**. Abbreviations are as follows: S represents superior, I represents inferior, N represents nasal, T represents temporal.

After MEMRI examination, retinal whole mounts were prepared to evaluate the ganglion cell layer expression of Chop2-GFP of all virus-injected eyes. As expected all eyes showed robust expression of GFP in ganglion cell somas, dendrites, and axons, with regions of high expression levels close to the optic nerve head (Figure 1A). In this restricted region GFP fluorescence appeared symmetrically and evenly distributed about the optic nerve (Figure 1B-H; white rectangular) in both dark-adapted (Figure 1B-D) and light-exposed (Figure 1E-H) retinas.

**Figure 2:**
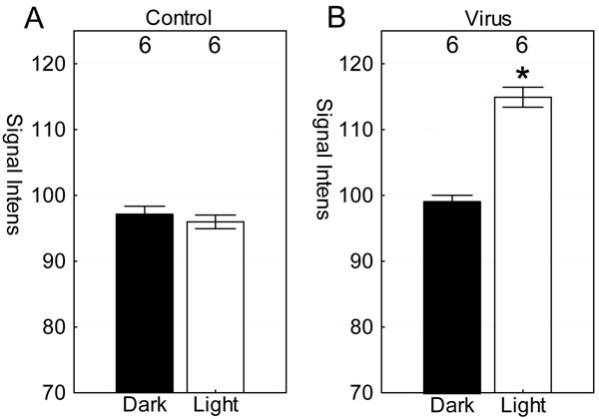
Horizontal analysis of the retina in light and dark conditions. Retinal signal intensities were measured in combined control groups (i.e., from noninjected and saline injected animals; (**A**) and in virus-injected mice (**B**). The numbers of animals used to generate these data are listed above each group. Error bars represent the standard error of the mean. *, p<0.05 was considered significant for the between-group comparison.

Similar restricted retinal regions as those described above were analyzed from the MEMRI data (±0.4–1 mm from the optic nerve head in superior–inferior directions; 620 μm slice thickness). Signal intensities of light-exposed, virus-treated eyes were not different (p>0.05) between superior (mean±SEM 113.4±2.1 arbitrary units [a.u.]) and inferior (116.4±2.2 a.u.) retina; these regions were combined for further analysis. Horizontal signal intensities were not different (p>0.05) between the two control groups (i.e., uninjected and saline-injected groups), regardless of lighting condition (Figure 2); these data were pooled for additional comparison. Also, no difference (p>0.05) was found between the combined control signal intensity and that in dark-exposed virus-treated eyes (Figure 2). Signal intensities from light-exposed virus-treated eyes were significantly different (p<0.05) compared to each of the other groups (Figure 2).

**Figure 3:**
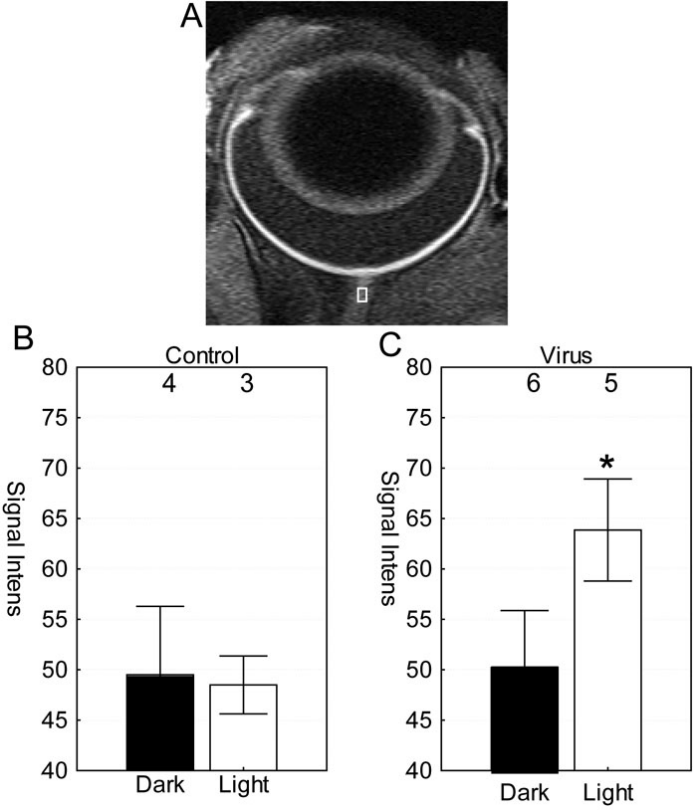
Optic nerve analysis in light and dark conditions. **A**: Region-of-interest on optic nerve of light-exposed ChR2-infected eye is shown in the white box. Optic nerve signal intensities were measured in the combined control groups (i.e., from noninjected and saline-injected animals; (**B**) and in the virus-injected mice (**C**). The numbers of animals used to generate these data are listed above each group. Error bars represent the standard error of the mean. *, p<0.05 was considered significant for the comparison between light-exposed Chop2-GFP eyes and combined control groups as well as that of the combined control groups and dark-exposed Chop2-GFP .

In the retinal optic nerve, no differences (p>0.05) in retinal manganese accumulation were found between the noninjected and saline-injected eyes regardless of lighting condition (Figure 3), and the data were combined. The combined control intensities (49.1±3.8 a.u., n=7) were similar to that of dark-exposed virus-treated eyes (50.2±5.7 a.u., n=6). For comparison, light-exposed virus-treated eyes had a greater (p<0.05) optic nerve signal relative to that of the combined control groups as well as that of the combined control groups and dark-exposed Chop2-GFP (Figure 3).

### Vertical analysis

**Figure 4:**
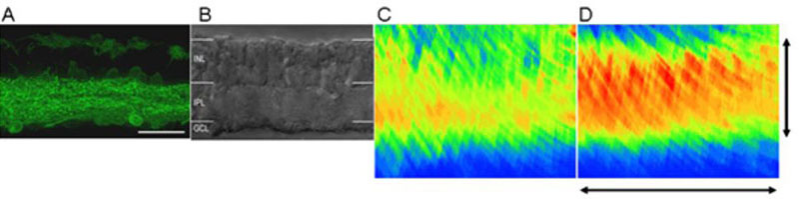
Vertical analysis of the retina. **A**: Numerous green fluorescent protein (GFP)-positive ganglion, amacrine, and horizontal cells can be seen in the cryostat section of a retina after a light-exposed magnetic resonance imaging experiment. **B**: Nomarski image of the same region shown in (**A**) reveals the absence of photoreceptors. Abbreviations are as follows: INL represents inner nuclear layer; IPL represents inner plexiform layer; GCL represents ganglion cell layer. The scale bar is equal to 25 μm in **A** and **B**. **C-D**: Pseudocolor linearized average 1/T1 maps of the central retina from uninjected (**C**) and virus injected (**D**) eyes, scaled vertically to approximately match the boundaries of (**A**) and (**B**). The pseudocolor scale was *blue* to *green* to *yellow* to *red*, which represents lowest (0.56 s^−1^) to highest (2.25 s^−1^) 1/T_1_ values. 1/T_1_ is directly proportional to manganese concentration [16]. The vertical scale bar is equal to 130 μm, and the horizontal scale bar is 0.6 mm and applies to **C** and **D**.

**Figure 5:**
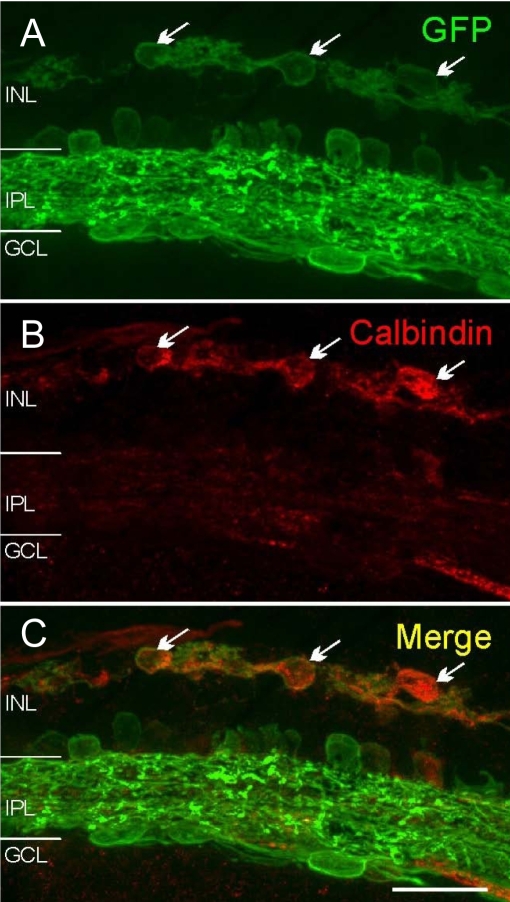
Double labeling of a retina after light-exposed magnetic resonance imaging experiment. Numerous green fluorescent protein (GFP)-positive cells (arrows, **A**) were immunolabelled for calbindin (**B**), a marker of horizontal cells. Merge image is shown in **C**. These data do not suggest that there were substantial numbers of infected remodeled cones (which would not be stained for calbindin). Abbreviations are as follows: INL represents inner nuclear layer; IPL represents inner plexiform layer; GCL represents ganglion cell layer. The scale bar is equal to 25 μm.

After MEMRI experiments and subsequent inspection in whole mounts, the Chop2-GFP-expressing retinas were further investigated in cryostat sections. The Chop2-GFP fluorescence was predominantly observed in the inner plexiform layer and in the ganglion cell layer (Figure 4A). In the INL, some AII amacrine cells were also labeled [3]. GFP-positive cells at the outer border of the INL are horizontal cells (based on double labeling with the antibody against calbindin, Figure 5). No glial cells with GFP fluorescence were detected. In the same retina vertical section, differential interference contrast images are consistent with the absence of photoreceptors (Figure 4B).

For MEMRI we collected the signals in the areas between ±0.4–1 mm on superior and inferior sides of the optic nerve. The signal was averaged over 600 μm retinal thickness (Figure 4C,D) and thus represents more partial volume averaging than that shown in Figure 4A,B. Relative to noninjected eyes (Figure 4C), light-exposed virus-infected eyes (Figure 4D) accumulated more (p<0.05) manganese in the retinal ganglion cells, inner plexiform, and inner nuclear layers (INLs).

## Discussion

In this study the major findings are that *rd1/rd1* mice with Chop2-GFP expression and exposure to bright blue light had 1) a horizontal distribution of manganese and Chop2-GFP expression that were symmetric about the optic nerve, 2) retinal and optic nerve accumulation of manganese that was greater than that measured in a series of control conditions, and 3) vertical loci of manganese accumulation and Chop2-GFP expression that were largely similar, except possibly in the INL (*vide infra*). These findings demonstrate for the first time the feasibility of using MEMRI to measure regional light responses in Chop2-GFP-expressing retinas in vivo.

The *rd1/rd1* mouse [22,23] is a widely used photoreceptor degeneration model that undergoes rapid retinal degeneration due to a defect in the β-subunit of a rod-specific cyclic guanosine monophosphate (cGMP)- phosphodiesterase and lacks the entire photoreceptor layer in adulthood [22]. The rd mutation initially affects rod photoreceptors, followed by a slower degradation of cones. By 4 months of age only approximately 1.3% of cones are left in the central retina [24,25]. In this study, the observed light- and dark-induced changes in the surviving retina measured (at the age of 4–5 months old) could be manifested by remaining cones [24,25], endogenous melanopsin [26], and/or exogenously introduced ChR2 [1]. However, it seems unlikely that melanopsin or the remaining cones are playing a role in the present study. In the two non-virus-injected control groups, no change in manganese uptake between light and dark conditions were found. Also, unlike the newborn retina, in the adult retina of wild-type and *rd1/rd1* mice, melanopsin is normally present in only a relatively small number of specialized ganglion cells [27,28]. With these considerations and given the large ectopic expression of ChR2 in this study (Figure 1), it is reasonable to conclude that ChR2 expression dominates the observed light/dark effect.

Previous evidence indicated that the intensity of the bright blue light used herein is sufficient to activate ChR2 in the retina in vivo [3,6]. As shown in Figure 2, such blue light exposure after ChR2 treatment also significantly increased manganese uptake. To the best of our knowledge, it is not yet known if manganese ions directly permeate the ChR2 channel. However, Mn^2+^ can act as a Ca^2+^ surrogate, and calcium ions are reported to permeate the ChR2 channel [2,29]. An alternative and not mutually exclusive possibility is that a light-induced opening of the ChR2 channel causes depolarization of the cell membrane, which activated other membrane conductances, including L-type voltage-gated calcium channels. L-type voltage-gated calcium channels are expressed throughout the rodent retina [30]. Since manganese readily permeates L-type channels, we speculate that this indirect ChR2-mediated route would be, at least in part, involved in the manganese uptake [11,29].

In light-exposed eyes, reasonable agreement was found between retinal and optic nerve manganese accumulation and Chop2-GFP expression and in the inner half of the surviving retina (i.e., the ganglion cell and inner plexiform layers). Although focal detection of Chop2-GFP-positive amacrine and horizontal cells was found in the INL, the 1/T_1_ maps suggested a somewhat greater involvement of the entire outer half of the remaining retina—corresponding to the INL—of the light-exposed virus-injected eyes (Figure 4D).

In the light-exposed virus-treated eyes, depolarization of the neurons in the INL with subsequent manganese accumulation might be possible. For example, the Chop2-GFP expressing amacrine cells (Figure 4A), together along with their downstream ON cone bipolar cells could depolarize during light illumination [31]. In addition, with light exposure horizontal cells expressing Chop2-GFP (Figure 4A) may depolarize, releasing gamma-aminobutyric acid (GABA). Rod bipolar cells in *rd1/rd1* mice retain functional dendritic GABA receptors [32]. In wild-type retinas GABA leads to depolarization of ON bipolar cells [33]. Assuming that the chloride gradient in bipolar cells of *rd1/rd1* mice is comparable to that of wild-type mice, ChR2-mediated depolarization of horizontal cells could also lead to depolarization of ON bipolar cells. These considerations support the idea that, in response to light, ChR2-expressing, amacrine, and horizontal cells can lead to depolarization of ON bipolar cells, with the increased possibility of greater manganese entry and accumulation. While the present resolution of the current MEMRI data (23.4 μm) was sufficient in 1/T_1_ maps of uninjected eyes to separate the inner from the outer half of the surviving retina, it may be inadequate for finer discrimination of histological sublamina. Also, while image registration and linearization were reasonably accurate, some subtle reductions in the effective resolution of retinal structure are likely. The distance between AII amacrine cells in the inner portion of the INL and the horizontal cells in the outer portion of the INL is about 20 μm. For these reasons, in the light-exposed virus-injected retinal 1/T_1_ map (Figure 4D), the absence of a “gap” in the space corresponding to mid-INL should not be construed as definitive evidence that bipolar cells show light-dependent depolarization in ChR2-expressing retinas. More work, with higher spatial resolution, is needed to test the reasonableness of these ideas.

In summary, first-time evidence is presented that demonstrates the sensitivity of MEMRI to regional light responses in Chop2-GFP-expressing retinas. More work is now needed to analytically evaluate how the spatial distribution and extent of retinal manganese uptake are linked, for example, with light exposure frequency and intensity. Nonetheless, these data raise the possibility that MEMRI will be useful in analytically evaluating the activity of ChR2, or its derivatives, or other microbial rhodopsins in the retina [34,35]. Thus, MEMRI is expected to facilitate the development and evaluation of the ChR2-based gene therapy for treating retinal degenerative diseases.
